# ﻿*Quercusmangdenensis*, a new species of *Quercus* (Fagaceae) from Kon Tum Province, Vietnam

**DOI:** 10.3897/phytokeys.215.93684

**Published:** 2022-12-15

**Authors:** Nguyen Van Ngoc, Hoang Thi Binh

**Affiliations:** 1 Faculty of Biology, Dalat University, 01- Phu Dong Thien Vuong, Dalat, Vietnam Dalat University Dalat Vietnam

**Keywords:** Fagales, flora, Kon Plong, Mang Den, taxonomy

## Abstract

*Quercusmangdenensis* Binh & Ngoc, **sp. nov.** (Fagaceae) is newly described from Mang Den Town in the central highland of Vietnam. The new species is characterized by lanceolate to oblong-lanceolate leaves with entire margin, 1–5-fruited infructescence, larger fruit size 6–10.5 cm long, broadly bowl-shaped cupules enclosing 1/5 of the nut, bracts of cupule entire and arranged in 5–7 rings, and cylindrical-ellipsoid and basally flat nuts 4.5–7.5 cm long. *Quercusmangdenensis* is morphologically similar to *Q.bidoupensis* Binh & Ngoc and *Q.kontumensis* A.Camus in having similar leaf shape, cuneate leaf base, and bracts arrangement in cupules. However, it differs from *Q.bidoupensis* and *Q.kontumensis* by cupules broadly bowl-shaped, much larger fruits, cylindrical-ellipsoid nut shape, and cupule enclosing 1/5 of the nuts. A description, photographs, and preliminary species conservation status of the new species are also provided.

## ﻿Introduction

*Quercus* L. is the biggest genus of the family Fagaceae, comprising more than 500 species widely distributed in Europe, North America, the Mediterranean, temperate deciduous forests in East Asia, and tropical montane forests in Southeast Asia (Nixon 1993; Huang et al. 1999; Phengklai 2008; Hubert et al. 2014; Valencia-A et al. 2016). In Vietnam, a total of 52 *Quercus* species have been reported (Ho 2003; Ban 2005; Li et al. 2016; Binh et al. 2018a, b, c; Binh et al. 2021; Ngoc et al. 2022), of which, recent efforts in taxonomic works of the genus *Quercus* have resulted in the description of seven new species from Vietnam (Binh et al. 2018a, b, c; Binh et al. 2021; Ngoc et al. 2022), indicating that the species diversity of *Quercus* of the country is significant. Therefore, taxonomic studies of the Vietnamese *Quercus* are still required.

**Figure 1.**
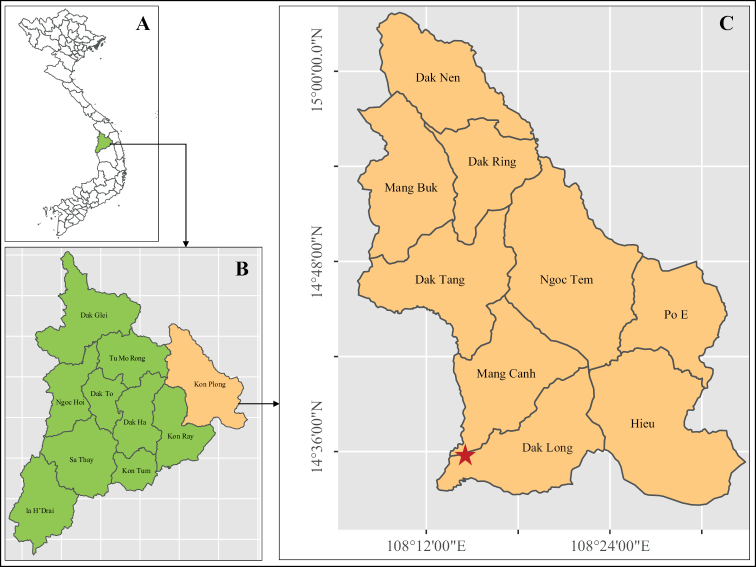
Type locality of *Quercusmangdenensis* Binh & Ngoc **A** map of Vietnam **B** map of Kon Tum province **C** map of Kon Plong District, the red star indicated the type locality: Mang Den Town, Dak Long Commune.

Mang Den town belongs to Kon Plong District, Kon Tum Province of Vietnam with a total area of 148.07 km^2^ (Kon Tum Province 2022) (Fig. 1). In this area, the annual temperature range is 18.7–24.9 °C and the average annual rainfall is around 1780–2200 mm with the rainy season from August to February (Tri et al. 2022). In the region, 273 woody plants have been recorded (265 angiosperms and 8 gymnosperm species). As for Fagaceae, 20 species have been recorded from Kon Plong District (Thanh et al. 2001).

From April to July 2022, we conducted botanical surveys in Mang Den and collected several individuals of the genus *Quercus* that could not be placed with any previously described species. After careful morphological examination and taxonomic review, here we describe those individuals as *Quercusmangdenensis* Binh & Ngoc along with morphological comparisons with the most similar species, photographs, and a preliminary conservation assessment of the new species.

## ﻿Materials and methods

### ﻿Plant materials

In this study, six specimens of the new species (*Binh et al. QC204, 205 206, 207, 208, 209*) were collected from Mang Den, Kon Plong District, Kon Tum Province (Fig. 1). In addition, we used type specimens and specimens that were collected from the type locality of the following species: *Q.bidoupensis* (*Tagane et al. V4328* [DLU!], *Binh et al. QC27, 29, 30, 45, 57* [DLU!]) and *Q.kontumensis* (*Poilane 18381* [P00754004] and [P00754005]), which are morphologically most similar to the new species in having a lanceolate to oblong-lanceolate leaf shape, cuneate leaf base, and bracts of cupule fusing in 5–7 rings.

### ﻿Morphological observations

To confirm that the species was undescribed, the relevant literature including Camus (1934), Soepadmo (1972), Huang et al. (1999), Ho (2003), Ban (2005), and Phengklai (2008), Binh et al. 2018a, b, c; Binh et al. 2021; Ngoc et al. 2022 were consulted. Then, we examined specimens in the herbaria at DLU, K, P, and VNM as well as digital images on the websites of JSTOR Global Plants (https://plants.jstor.org/) and the Chinese Virtual Herbarium (http://www.cvh.ac.cn/).

## ﻿Taxonomic treatment

### ﻿ Quercus mangdenensis

Taxon classificationPlantaeFagalesFagaceae

H.T.Binh & Ngoc sp. nov.

077082FA-4BD4-58FA-A6F7-565B75B40D28

urn:lsid:ipni.org:names:77309987-1

Fig. 2

#### Diagnosis.

*Quercusmangdenensis* is morphologically most similar to *Q.bidoupensis* and *Q.kontumensis* with a lanceolate to oblong-lanceolate leaf shape, cuneate leaf base, and bracts of cupule fusing in 5–7 rings. However, *Q.mangdenensis* is distinguished from *Q.bidoupensis* by its entire leaf margin (vs. undulate, distinctly serrate in the upper 1/2), longer petioles 2.5–4 cm long (vs. 1.3–2 cm long), broadly bowl-shaped cupules (vs. obconical-shaped), larger cupule (2.6–3.5 cm high × 4–5 cm in diam. vs. 1.3–1.5 cm high × 1.3–1.7 cm in diam.), enclosure of cupule (1/5 vs. 1/3 of the nut), cylindrical-ellipsoid nut (vs. ovoid), larger nut (4.5–7.5 cm high × 3–4 cm in diam. vs. 2.2 cm high × 1.4 cm in diam.), and flat basal scar of the nut (vs. convex). It differs from *Q.kontumensis* in having larger cupule enclosing 1/5 of the nut (2.6–3.5 cm high × 4–5 cm in diam. vs. 1–1.2 cm long × 1.5–2 cm in diam., enclosing 1/2 of the nut), larger cylindrical-ellipsoid nut 4.5–7.5 cm high × 3–4 cm in diam. (vs. cylindrical-ovoid, 2 cm high × 1.5 cm in diam.), and scales of cupule fusing into 5–7 entire ridges (vs. undulate) (Table 1).

**Table 1.** Morphological comparison amongst *Quercusmangdenensis* Binh & Ngoc, sp. nov., *Quercusbidoupensis* Binh & Ngoc and *Quercuskontumensis* A. Camus.

Characters	*Q.mangdenensis*	*Q.bidoupensis* ^(1,2)^	*Q.kontumensis* ^(3,4)^
Buds shape	gobose to oblate	oblong to ellipsoid	ovoid to ellipsoid
Leaf shape	lanceolate to oblong-lanceolate	oblong-lanceolate	lanceolate to oblong-lanceolate
Leaf margin	entire	margins undulate, distinctly serrate in the upper 1/2	entire or distinctly serrate in the upper 1/5
Length of petioles	(1.3–)2.5–4 cm long	1.3–2 cm long	2.5–4.2 cm long
Number of secondary veins	(5–)7–9 pairs	10–13 pairs	5–8 pairs
Cupule shape and size	broadly bowl-shaped, 2.6–3.5 cm high, 4–5 cm in diam.	obconical-shaped, 1.3–1.5 cm high, 1.3–1.7 cm in diam.	cup-shaped, 1–1.2 cm long, 1.5–2 cm in diam.
Number of rings on cupule	5–6 rings	5–6 rings	6–7 rings
Cupule bract margin	entire	entire	undulate
Cupule coverage	enclosing 1/5 of the nut	enclosing 1/3 of the nut	enclosing 1/2 of the nut
Nut shape and size	cylindrical-ellipsoid, 4.5–7.5 cm high, 3–4 cm in diam.	ovoid, 2.2 cm high, 1.4 cm in diam.	cylindrical-ovoid, 2 cm high, 1.5 cm in diam.
Base of the nut	Flat	Convex	Flat

^(1)^ From the protologue (Binh et al. 2018b); ^(2)^ From *Binh et al*. *QC27* (DLU); ^(3)^ From Camus (1934); ^(4)^ From the material *E. Poilane 18381* (P [P00754005, P00754005]).

**Figure 2.**
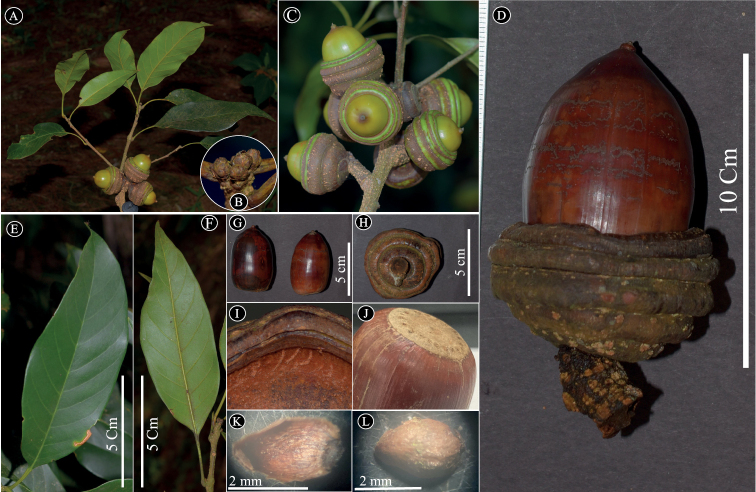
*Quercusmangdenensis* Binh & Ngoc: **A** twigs with young fruit **B** terminal buds **C** infructescences **D** fallen mature fruit **E, F** adaxial and abaxial surface of the leaves **G** nuts **H** outside of cupule **I** densely reddish hairs inside of cupules **J** basal scar of the nut **K, L** inside and outside of bud scale. Materials from *H.T. Binh and N.V. Ngoc QC204*.

#### Type.

Vietnam. Kontum Province, Kon Plong District, Mang Den Town, alt. 1179 m, 14°36'39.1"N, 108°15'00.5"E, 23 July 2022, *H.T. Binh & N.V. Ngoc QC204* (holotype DLU!; isotypes HN!, VNM!)

#### Description.

Tree, evergreen, 20–25 m tall, 60–80 cm girth. Bark whitish gray. Buds perulate, globose to oblate, 2.5–4 mm high, 2.5–3.5 mm in diam., bud scales imbricate, in 2–3 rows, ovate-triangular, 2.0–3.0 × 1.5–2.0 mm, apex obtuse, margin ciliate, covered with appressed white hairy outside, glabrous inside. Young twigs dark pale green, hairy, old twigs grayish-brown, glabrous, sometimes sulcate, lenticellate. Leave alternate; blades thickly coriaceous, lanceolate to oblong-lanceolate, (5.5–)11–16.5(–19.5) × (2.6–)3.5–5.5 cm, acute at apex, broadly cuneate at base, margin entire, glossy green adaxially, bright green abaxially, conspicuously pale creamy brown to yellowish brown *in sicco*, glabrous on both surfaces, midrib slightly raised on the upper surface, prominently raised on the lower surface, lateral veins (5–)7–9 pairs, prominent on the lower surface, at an angle of 45–55 degree from the midrib, straight and then curved near margin, tertiary veins scalariform-reticulate, faintly visible on both surfaces; petioles (1.3–)2.5–4 cm long, yellowish brown when dry, glabrous. Male and female inflorescences not seen. Infructescences axillary or pseudo-terminal (sometimes in upper leaf-scars), erect, rachis 1–1.5 cm long, 1–1.3 cm in diam., woody, glabrous, dark yellow *in vivo*, yellowish brown *in sicco*, lenticellate. Mature fruits 6–10.5 cm high (including cupule), solitary, sessile on woody rachis; nuts cylindrical-ellipsoid, 4.5–7.5 cm high, 3–4 cm in diam., rounded at the top, white tomentose around stylopodia, stylopodia 1.5–2.5 mm long, basal scar 2–2.5 cm in diam., flat; cupules broadly bowl-shaped, 2.6–3.5 cm high, 4–5 cm in diam., enclosing 1/5 of the nut when mature, outside tomentose with reddish hairs to glabrous, inside with appressed densely reddish hairs, wall 0.5–1.3 cm thick, woody, bracts fusing and arranged in 5–7 ridges (rings), the rings’ margin completely entire (without scale-like structure).

#### Distribution.

Vietnam. Kon Tum Province, Kon Plong District, Mang Den Town (Fig. 1).

#### Habitat.

*Quercusmangdenensis* were found in the scattered evergreen forest, from 1050 to 1200 m elevation.

#### Etymology.

The specific epithet is derived from its type locality, Mang Den Town, Kon Plong District, Kon Tum Province, Vietnam.

#### Vernacular name.

Sồi Măng Đen (suggested here).

#### Phenology.

Fruiting specimens and fallen mature fruits were collected in July.

#### Additional specimens examined.

Vietnam. Kontum Province, Kon Plong District, Mang Den, alt. 1168 m, 14°36'37.4"N, 108°15'11.6"E, 23 July 2022, *H.T. Binh & N.V. Ngoc QC205* [fr.] (DLU!, HN!, VNM!); ibid., 1180 m elev., 14°36'32.5"N, 108°15'18.2"E, 23 July 2022, *H.T. Binh & N.V. Ngoc QC207* [fr.] (DLU!, HN!, VNM); ibid., 1130 m elev., 14°36'36.5"N, 108°15'31.3"E, 23 July 2022, *H.T. Binh & N.V. Ngoc QC208* [fr.] (DLU!, HN!, VNM); ibid., 1065 m elev., 14°36'36.5"N, 108°15'31.3"E, 23 July 2022, *H.T. Binh & N.V. Ngoc QC209* [fr.] (DLU!, HN!, VNM!).

#### Preliminary conservation status.

Our botanical inventories were conducted at Mang Den and the surrounding areas from April to July 2022 and we found five subpopulations of 5–7 mature individuals of *Quercusmangdenensis* in the scattered evergreen forests. In addition, the forest in this area is severely fragmented and continuing to decline caused by human activities, such as farming, logging, and harvesting of non-timber forest products, etc., while almost all of the individuals of *Q.mangdenensis* are distributed along the boundary between the evergreen forest and local people’s farms. According to criterion D of the IUCN Red List criteria (IUCN 2019), the species classifies as Critically Endangered (CR). The area of occupancy (AOO) and the extent of occurrence (EOO) were calculated are 0.415 km^2^ and 8.0 km^2^, respectively. Based on criterion B of the IUCN Red List criteria (IUCN 2019), the new species is qualified as Critically Endangered [CR B1ab(i,ii,iii,iv,v) B2ab(i,ii,iii,iv,v)].

## Supplementary Material

XML Treatment for Quercus mangdenensis
